# 4-Methyl-*N*-(4-methyl­phenyl­sulfon­yl)-*N*-phenyl­benzene­sulfonamide

**DOI:** 10.1107/S1600536814002086

**Published:** 2014-02-05

**Authors:** Bilge Eren, Selçuk Demir, Hakan Dal, Tuncer Hökelek

**Affiliations:** a Department of Chemistry, Bilecik Şeyh Edebali University, 11210 Bilecik, Turkey; b Department of Chemistry, Recep Tayyip Erdoğan University, 53100 Rize, Turkey; c Department of Chemistry, Anadolu University, 26470 Eskişehir, Turkey; d Department of Physics, Hacettepe University, 06800 Beytepe, Ankara, Turkey

## Abstract

The whole mol­ecule of the title compound, C_20_H_19_NO_4_S_2_, is generated by twofold rotational symmetry. The N atom is located on the twofold rotation axis and has a trigonal-planar geometry. It is bonded by two S atoms of two symmetry-related 4-methyl­phenyl­sulfonyl groups and by the C atom of the phenyl ring, which is bis­ected by the twofold rotation axis. The benzene and phenyl rings are oriented at a dihedral angle of 51.48 (5)° while the pendant benzene rings are inclined to one another by 87.76 (9)°. In the crystal, weak C—H⋯O hydrogen bonds link the mol­ecules, forming a three-dimensional network.

## Related literature   

Several sulfonamide derivatives have been used as chemotherapeutic agents for their anti­bacterial, anti­fungal, anti­tumor and hypoglycemic effects for many years, see: Chohan *et al.* (2010 ▶); El-Sayed *et al.* (2011 ▶); Seri *et al.* (2000 ▶). Some sulfonamide derivatives are reported to have carbonic anhydrases (CA) inhibition properties, see: Suparan *et al.* (2000 ▶). For the use of disulfonamides for their anti­tumor activity and CA inhibitory properties, see: Boriack-Sjodin *et al.* (1998 ▶). For the use as catalysts in asymmetric syntheses of complexes obtained from disulfonamides chiral derivatives, see: Guo *et al.* (1997 ▶). For sulfonation of aniline by 4-tolyl­sulfonyl chloride utilizing standard procedures with small modifications, see: DeChristopher *et al.* (1974 ▶). For a related structure involving 4-methyl­phenyl­sulfonyl, see: Elgemeie *et al.* (2013 ▶). For bond-length data, see: Allen *et al.* (1987 ▶).
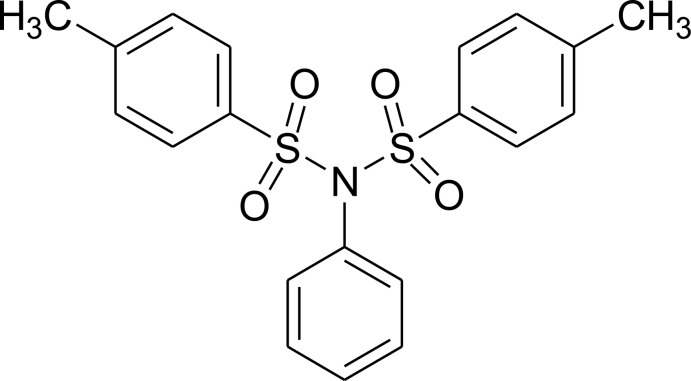


## Experimental   

### 

#### Crystal data  

C_20_H_19_NO_4_S_2_
*M* *_r_* = 401.51
Monoclinic, 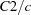

*a* = 18.1080 (5) Å
*b* = 9.3834 (3) Å
*c* = 11.4821 (4) Å
β = 96.015 (3)°
*V* = 1940.24 (11) Å^3^
*Z* = 4
Mo *K*α radiation
μ = 0.30 mm^−1^
*T* = 296 K
0.25 × 0.22 × 0.14 mm

#### Data collection  

Bruker Kappa APEXII CCD area-detector diffractometer
9411 measured reflections
2441 independent reflections
1981 reflections with *I* > 2σ(*I*)
*R*_int_ = 0.022

#### Refinement  

*R*[*F* ^2^ > 2σ(*F* ^2^)] = 0.038
*wR*(*F* ^2^) = 0.109
*S* = 1.05
2441 reflections
125 parameters
H-atom parameters constrained
Δρ_max_ = 0.24 e Å^−3^
Δρ_min_ = −0.39 e Å^−3^

### 

Data collection: *APEX2* (Bruker, 2007 ▶); cell refinement: *SAINT* (Bruker, 2007 ▶); data reduction: *SAINT*; program(s) used to solve structure: *SHELXS97* (Sheldrick, 2008 ▶); program(s) used to refine structure: *SHELXL97* (Sheldrick, 2008 ▶); molecular graphics: *ORTEP-3 for Windows* (Farrugia, 2012 ▶); software used to prepare material for publication: *WinGX* (Farrugia, 2012 ▶) and *PLATON* (Spek, 2009 ▶).

## Supplementary Material

Crystal structure: contains datablock(s) I, global. DOI: 10.1107/S1600536814002086/su2692sup1.cif

Structure factors: contains datablock(s) I. DOI: 10.1107/S1600536814002086/su2692Isup2.hkl

Supporting information file. DOI: 10.1107/S1600536814002086/su2692Isup3.cml

CCDC reference:

Additional supporting information:  crystallographic information; 3D view; checkCIF report

## Figures and Tables

**Table 1:** Hydrogen-bond geometry (Å, °)

*D*—H⋯*A*	*D*—H	H⋯*A*	*D*⋯*A*	*D*—H⋯*A*
C6—H6⋯O2^i^	0.93	2.59	3.337 (2)	138
C9—H9⋯O1^ii^	0.93	2.58	3.496 (2)	168
C10—H10⋯O2^iii^	0.93	2.59	3.408 (3)	147
C10—H10⋯O2^iv^	0.93	2.59	3.408 (3)	147

Symmetry codes: (i) 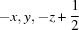; (ii) 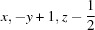; (iii) 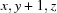; (iv) 
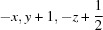
.

**Fig. 1.**
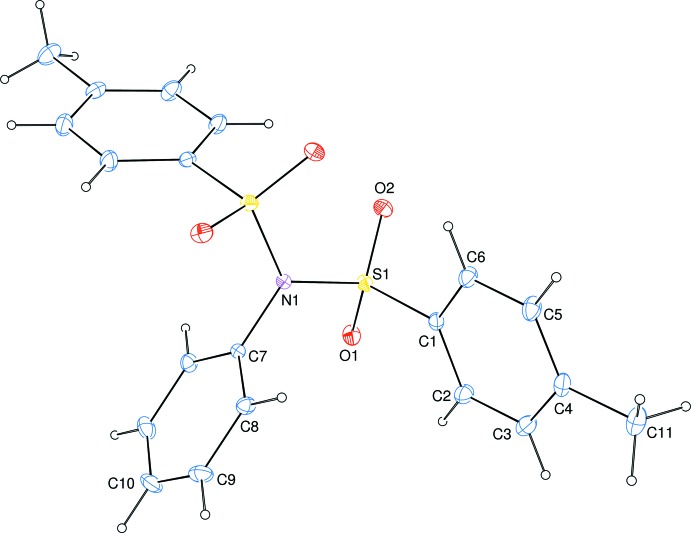
A view of the molecular structure of the title molecule, with atom-labelling. Displacement ellipsoids are drawn at the 50% probability level. The two-fold rotation axis bisects atoms N1, C7 and C10.

**Fig. 2.**
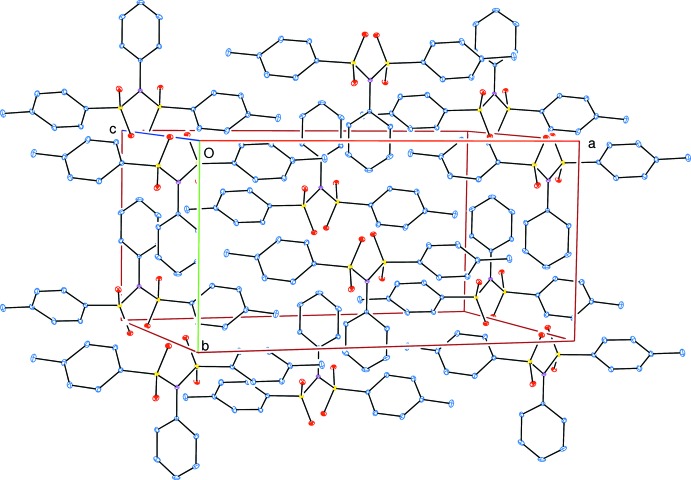
A partial view along the c axis of the crystal packing of the title compound. Hydrogen atoms have been omitted for clarity.

## supplementary crystallographic information

### 1. Comment

Sulfonamides, which are known as sulfa drugs, are an important class of compounds in the field of chemistry and pharmacology. Several sulfonamide derivatives are used as chemotherapeutic agents for their antibacterial, antifungal, antitumor and hypoglycemic (Chohan *et al.*, 2010; El-Sayed *et al.*, 2011; Seri *et al.*, 2000). In addition, some sulfonamide derivatives are reported to have carbonic anhydrases (CA) inhibition properties (Suparan *et al.*, 2000). Disulfonamides are sulfonamide derivatives containing two sulfone groups connected to the nitrogen atom and they are used for their antitumor activity and CA inhibitory properties (Boriack-Sjodin *et al.*, 1998). On the other hand, the complexes obtained from their chiral derivatives are used in asymmetric syntheses as catalysts (Guo *et al.*, 1997). The title compound, belonging to the disulfonimide group, was synthesized and its crystal structure is reported herein.

The asymmetric unit of the title compound contains half a molecule; the whole molecule is generated by two-fold rotational symmetry. Atoms N1, C7 and C10 are located on the two-fold rotation axis (Fig. 1). The geometry around atoms S1 and N1 are distorted tetrahedral and planar trigonal, respectively. The average S—O bond length is 1.4213 (13) Å, while the S—N and S—C bond lengths are 1.6822 (9) and 1.7546 (17), respectively. These distances are close to standard values (Allen *et al.*, 1987) and may be compared with the corresponding values in 5-amino-1-(4-methylphenylsulfonyl)-4-pyrazolin-3-one (Elgemeie *et al.*, 2013). The benzene and phenyl rings are oriented at a dihedral angle of 51.48 (5)°. Atoms S1, C11 and N1 are displaced by -0.0757 (4), -0.0172 (26) and -0.0018 (1) Å from the adjacent ring planes.

In the crystal, Fig. 2, weak C—H···O hydrogen bonds (Table 1) link the molecules into a three-dimensional network.

### 2. Experimental

The title compound was prepared by a two step sulfonylation of aniline by 4-toluenesulfonyl chloride utilizing standard procedures with small modifications (DeChristopher *et al.*, 1974). Aniline (40 mmol) and benzene (10 ml) were placed in a two-necked flask fitted with a dropping funnel and a reflux condenser. A solution of 4-toluenesulfonyl chloride (20 mmol) in benzene (50 ml) was placed in the dropping funnel and was added to the aniline solution in portions with stirring. The mixture was heated under reflux for 2 h. The obtained heterogeneous mixture was cooled and the solvent was evaporated under vacuum. The crude product was treated sequentially with deionized water (20 ml) and NaOH (20%) solution. The mixture was placed in a separation funnel and the water phase was separated and acidified gently with 4M HCl. The precipitate 4-toluene sulfonanilide was collected by filtration, and then dried (yield: 3.66 g, 74%; m.p. 372-373 K). In the second step 4-toluene sulfonanilide (10 mmol) was dissolved in benzene (30 ml) and the mixture stirred under reflux. A solution of 4-toluenesulfonyl chloride (10 mmol) in benzene (30 ml) was added drop wise into the stirring solution, and then potassium tert-butoxide (12 mmol), followed by catalytic amounts of 18-crown-6 were added in portions. After the reaction system was allowed to reflux for 3 h, then the mixture was cooled and the solvent evaporated under vacuum. The crude product was treated with NaOH (20%) solution in order to remove excess 4-toluene sulfonanilide. The insoluble solids were collected by filtration, washed with deionized water, and then dried (yield: 3.21 g, 78%; m.p. 454-456 K). The suitable colourless block-like crystals were obtained by recrystallization from acetone/water (7:3).

### 3. Refinement

The C-bound H-atoms were positioned geometrically with C—H = 0.93 and 0.96 Å for aromatic and methyl H-atoms, respectively, and constrained to ride on their parent atoms, with *U*_iso_(H) = k × *U*_eq_(C), where k = 1.5 for methyl H-atoms and = 1.2 for other H-atoms.

### Crystal data

**Table:**

C_20_H_19_NO_4_S_2_	*F*(000) = 840
*M**_r_* = 401.51	*D*_x_ = 1.375 Mg m^−^^3^
Monoclinic, *C*2/*c*	Mo *K*α radiation, λ = 0.71073 Å
Hall symbol: -C 2yc	Cell parameters from 3887 reflections
*a* = 18.1080 (5) Å	θ = 2.3–28.1°
*b* = 9.3834 (3) Å	µ = 0.30 mm^−^^1^
*c* = 11.4821 (4) Å	*T* = 296 K
β = 96.015 (3)°	Block, colourless
*V* = 1940.24 (11) Å^3^	0.25 × 0.22 × 0.14 mm
*Z* = 4	

### Data collection

**Table:**

Bruker Kappa APEXII CCD area-detector diffractometer	1981 reflections with *I* > 2σ(*I*)
Radiation source: fine-focus sealed tube	*R*_int_ = 0.022
Graphite monochromator	θ_max_ = 28.4°, θ_min_ = 2.3°
φ and ω scans	*h* = −24→23
9411 measured reflections	*k* = −10→12
2441 independent reflections	*l* = −15→15

### Refinement

**Table:**

Refinement on *F*^2^	Primary atom site location: structure-invariant direct methods
Least-squares matrix: full	Secondary atom site location: difference Fourier map
*R*[*F*^2^ > 2σ(*F*^2^)] = 0.038	Hydrogen site location: inferred from neighbouring sites
*wR*(*F*^2^) = 0.109	H-atom parameters constrained
*S* = 1.05	*w* = 1/[σ^2^(*F*_o_^2^) + (0.0574*P*)^2^ + 0.990*P*] where *P* = (*F*_o_^2^ + 2*F*_c_^2^)/3
2441 reflections	(Δ/σ)_max_ < 0.001
125 parameters	Δρ_max_ = 0.24 e Å^−^^3^
0 restraints	Δρ_min_ = −0.39 e Å^−^^3^

### Special details

**Table:**

Geometry. All esds (except the esd in the dihedral angle between two l.s. planes) are estimated using the full covariance matrix. The cell esds are taken into account individually in the estimation of esds in distances, angles and torsion angles; correlations between esds in cell parameters are only used when they are defined by crystal symmetry. An approximate (isotropic) treatment of cell esds is used for estimating esds involving l.s. planes.
Refinement. Refinement of F^2^ against ALL reflections. The weighted R-factor wR and goodness of fit S are based on F^2^, conventional R-factors R are based on F, with F set to zero for negative F^2^. The threshold expression of F^2^ > 2sigma(F^2^) is used only for calculating R-factors(gt) etc. and is not relevant to the choice of reflections for refinement. R-factors based on F^2^ are statistically about twice as large as those based on F, and R- factors based on ALL data will be even larger.

### Fractional atomic coordinates and isotropic or equivalent isotropic displacement parameters (Å^2^)

**Table:**

	*x*	*y*	*z*	*U*_iso_*/*U*_eq_	
S1	0.06888 (2)	0.13028 (4)	0.33345 (3)	0.03537 (14)	
O1	0.08971 (7)	0.22464 (13)	0.42817 (10)	0.0471 (3)	
O2	0.04424 (7)	−0.00962 (13)	0.35650 (11)	0.0491 (3)	
N1	0.0000	0.21329 (18)	0.2500	0.0332 (4)	
C1	0.14280 (9)	0.12114 (17)	0.24640 (14)	0.0369 (3)	
C2	0.20477 (10)	0.2045 (2)	0.27464 (17)	0.0533 (5)	
H2	0.2065	0.2682	0.3369	0.064*	
C3	0.26449 (11)	0.1920 (2)	0.2089 (2)	0.0622 (5)	
H3	0.3066	0.2472	0.2284	0.075*	
C4	0.26270 (10)	0.0998 (2)	0.11573 (17)	0.0502 (4)	
C5	0.19959 (10)	0.0185 (2)	0.08876 (17)	0.0555 (5)	
H5	0.1976	−0.0443	0.0258	0.067*	
C6	0.13953 (10)	0.0282 (2)	0.15261 (17)	0.0505 (4)	
H6	0.0974	−0.0270	0.1330	0.061*	
C7	0.0000	0.3675 (2)	0.2500	0.0303 (4)	
C8	0.03282 (9)	0.43935 (18)	0.16443 (14)	0.0416 (4)	
H8	0.0547	0.3897	0.1069	0.050*	
C9	0.03269 (12)	0.5865 (2)	0.16553 (18)	0.0575 (5)	
H9	0.0549	0.6366	0.1086	0.069*	
C10	0.0000	0.6588 (3)	0.2500	0.0642 (8)	
H10	0.0000	0.7579	0.2500	0.077*	
C11	0.32811 (12)	0.0864 (3)	0.0452 (2)	0.0711 (6)	
H11A	0.3110	0.0578	−0.0333	0.107*	
H11B	0.3620	0.0163	0.0805	0.107*	
H11C	0.3529	0.1767	0.0435	0.107*	

### Atomic displacement parameters (Å^2^)

**Table:**

	*U*^11^	*U*^22^	*U*^33^	*U*^12^	*U*^13^	*U*^23^
S1	0.0368 (2)	0.0329 (2)	0.0360 (2)	0.00350 (15)	0.00149 (15)	0.00050 (14)
O1	0.0507 (7)	0.0514 (7)	0.0377 (6)	0.0076 (6)	−0.0026 (5)	−0.0083 (5)
O2	0.0542 (7)	0.0365 (7)	0.0565 (7)	0.0010 (5)	0.0051 (6)	0.0118 (5)
N1	0.0307 (9)	0.0270 (9)	0.0416 (9)	0.000	0.0019 (7)	0.000
C1	0.0326 (7)	0.0355 (8)	0.0421 (8)	0.0054 (6)	0.0014 (6)	−0.0017 (6)
C2	0.0428 (10)	0.0543 (12)	0.0634 (11)	−0.0051 (8)	0.0085 (8)	−0.0180 (9)
C3	0.0400 (10)	0.0637 (14)	0.0836 (14)	−0.0079 (9)	0.0102 (9)	−0.0130 (11)
C4	0.0383 (9)	0.0586 (11)	0.0542 (10)	0.0136 (8)	0.0077 (8)	0.0061 (9)
C5	0.0433 (10)	0.0721 (13)	0.0507 (10)	0.0088 (9)	0.0036 (8)	−0.0189 (9)
C6	0.0373 (9)	0.0587 (11)	0.0551 (10)	0.0005 (8)	0.0023 (7)	−0.0181 (9)
C7	0.0289 (9)	0.0271 (10)	0.0353 (10)	0.000	0.0052 (8)	0.000
C8	0.0416 (9)	0.0435 (10)	0.0412 (8)	−0.0051 (7)	0.0116 (7)	0.0032 (7)
C9	0.0655 (13)	0.0455 (11)	0.0606 (11)	−0.0167 (10)	0.0013 (9)	0.0166 (9)
C10	0.076 (2)	0.0270 (13)	0.084 (2)	0.000	−0.0178 (17)	0.000
C11	0.0481 (11)	0.0943 (18)	0.0736 (14)	0.0158 (12)	0.0198 (10)	0.0051 (13)

### Geometric parameters (Å, º)

**Table:**

S1—O1	1.4223 (12)	C6—C5	1.377 (3)
S1—O2	1.4203 (13)	C6—H6	0.9300
S1—N1	1.6822 (9)	C7—N1	1.447 (3)
S1—C1	1.7546 (17)	C7—C8^i^	1.3768 (18)
N1—S1^i^	1.6822 (9)	C7—C8	1.3768 (18)
C1—C2	1.378 (2)	C8—C9	1.380 (3)
C1—C6	1.382 (2)	C8—H8	0.9300
C2—C3	1.387 (3)	C9—C10	1.368 (3)
C2—H2	0.9300	C9—H9	0.9300
C3—H3	0.9300	C10—C9^i^	1.368 (3)
C4—C3	1.374 (3)	C10—H10	0.9300
C4—C5	1.382 (3)	C11—H11A	0.9600
C4—C11	1.508 (3)	C11—H11B	0.9600
C5—H5	0.9300	C11—H11C	0.9600
			
O1—S1—N1	105.59 (7)	C6—C5—H5	119.2
O1—S1—C1	108.00 (8)	C1—C6—H6	120.5
O2—S1—O1	119.74 (8)	C5—C6—C1	118.97 (17)
O2—S1—N1	107.79 (8)	C5—C6—H6	120.5
O2—S1—C1	109.53 (8)	C8^i^—C7—N1	119.33 (10)
N1—S1—C1	105.22 (5)	C8—C7—N1	119.33 (10)
S1^i^—N1—S1	124.83 (11)	C8^i^—C7—C8	121.3 (2)
C7—N1—S1	117.58 (5)	C7—C8—C9	118.82 (17)
C7—N1—S1^i^	117.58 (5)	C7—C8—H8	120.6
C2—C1—S1	119.29 (13)	C9—C8—H8	120.6
C2—C1—C6	120.59 (16)	C8—C9—H9	119.9
C6—C1—S1	120.10 (13)	C10—C9—C8	120.26 (19)
C1—C2—C3	119.09 (17)	C10—C9—H9	119.9
C1—C2—H2	120.5	C9^i^—C10—C9	120.5 (3)
C3—C2—H2	120.5	C9^i^—C10—H10	119.8
C2—C3—H3	119.3	C9—C10—H10	119.8
C4—C3—C2	121.38 (18)	C4—C11—H11A	109.5
C4—C3—H3	119.3	C4—C11—H11B	109.5
C3—C4—C5	118.27 (17)	C4—C11—H11C	109.5
C3—C4—C11	120.97 (19)	H11A—C11—H11B	109.5
C5—C4—C11	120.76 (19)	H11A—C11—H11C	109.5
C4—C5—H5	119.2	H11B—C11—H11C	109.5
C6—C5—C4	121.70 (17)		
			
O1—S1—N1—S1^i^	−151.02 (6)	C2—C1—C6—C5	0.9 (3)
O1—S1—N1—C7	28.98 (6)	C1—C2—C3—C4	0.8 (3)
O2—S1—N1—S1^i^	−21.94 (6)	C5—C4—C3—C2	−0.2 (3)
O2—S1—N1—C7	158.06 (6)	C11—C4—C3—C2	−179.7 (2)
C1—S1—N1—S1^i^	94.88 (6)	C3—C4—C5—C6	−0.1 (3)
C1—S1—N1—C7	−85.12 (6)	C11—C4—C5—C6	179.42 (19)
O1—S1—C1—C2	−3.00 (17)	C1—C6—C5—C4	−0.3 (3)
O1—S1—C1—C6	175.15 (14)	C8—C7—N1—S1	94.21 (8)
O2—S1—C1—C2	−134.97 (15)	C8^i^—C7—N1—S1	−85.79 (8)
O2—S1—C1—C6	43.18 (16)	C8—C7—N1—S1^i^	−85.79 (8)
N1—S1—C1—C2	109.41 (15)	C8^i^—C7—N1—S1^i^	94.21 (8)
N1—S1—C1—C6	−72.44 (16)	N1—C7—C8—C9	−179.79 (12)
S1—C1—C2—C3	177.00 (16)	C8^i^—C7—C8—C9	0.21 (12)
C6—C1—C2—C3	−1.1 (3)	C7—C8—C9—C10	−0.4 (2)
S1—C1—C6—C5	−177.22 (15)	C8—C9—C10—C9^i^	0.21 (13)

Symmetry code: (i) −*x*, *y*, −*z*+1/2.

### Hydrogen-bond geometry (Å, º)

**Table:**

*D*—H···*A*	*D*—H	H···*A*	*D*···*A*	*D*—H···*A*
C6—H6···O2^i^	0.93	2.59	3.337 (2)	138
C9—H9···O1^ii^	0.93	2.58	3.496 (2)	168
C10—H10···O2^iii^	0.93	2.59	3.408 (3)	147
C10—H10···O2^iv^	0.93	2.59	3.408 (3)	147

Symmetry codes: (i) −*x*, *y*, −*z*+1/2; (ii) *x*, −*y*+1, *z*−1/2; (iii) *x*, *y*+1, *z*; (iv) −*x*, *y*+1, −*z*+1/2.
